# A nomogram for predicting severe adenovirus pneumonia in children

**DOI:** 10.3389/fped.2023.1122589

**Published:** 2023-03-01

**Authors:** Jiamin Zhang, Changdi Xu, Shasha Yan, Xuefang Zhang, Deyu Zhao, Feng Liu

**Affiliations:** Department of Respiratory Medicine, Children's Hospital of Nanjing Medical University, Nanjing, China

**Keywords:** adenovirus, pneumonia, early recognition scale, respiratory infection, risk facors

## Abstract

Adenoviral pneumonia in children was an epidemic that greatly impacted children's health in China in 2019. Currently, no simple or systematic scale has been introduced for the early identification and diagnosis of adenoviral pneumonia. The early recognition scale of pediatric severe adenovirus pneumonia was established based on an analysis of the children's community-acquired pneumonia clinical cohort. This study analyzed the clinical data of 132 children with adenoviral pneumonia who were admitted to the Children's Hospital of Nanjing Medical University. The clinical parameters and imaging features were analyzed using univariate and multivariate logistic regression analyses. A nomogram was constructed to predict the risk of developing severe adenovirus pneumonia in children. There were statistically significant differences in age, respiratory rate, fever duration before admission, percentage of neutrophils and lymphocytes, CRP, ALT, and LDH between the two groups. Logistic regression analysis was conducted using the R language, and respiratory rate, percentage of neutrophils, percentage of lymphocytes, and LDH were used as scale indicators. Using the ROC curve, the sensitivity and specificity of the scale were 93.3% and 92.1%. This scale has good sensitivity and specificity through internal verification, which proves that screening for early recognition of severe adenovirus pneumonia can be realized by scales. This predictive scale helps determine whether a child will develop severe adenovirus pneumonia early in the disease course.

## 1. Introduction

Adenovirus is an important pathogen that causes community-acquired pneumonia (CAP) in children (1). More than 80% of adenovirus pneumonia cases occur in children under the age of four, especially infants and young children <2 years old (2). Adenovirus pneumonia can develop in acute respiratory distress syndrome, critically ill patients with respiratory failure, toxic encephalopathy, hemophagocytic lymphohistiocytosis, post-infectious bronchiolitis obliterans (PIBO), and even death, and is an important cause of infant death and disability (3–6). Adenovirus is highly contagious in schools, hospitals, and other highly enclosed areas, and epidemics in crowded environments can occur (2).

Methods such as the Pneumonia Severity Index (PSI) score and CURB-65 score have been established in adult CAP to predict the severity and prognosis of adult CAP at an early stage and guide treatment (7, 8). Whereas, given the age limitations and practicality of these scales, they cannot be directly applied to children.

We previously developed a scale for the early prediction of refractory *Mycoplasma pneumoniae* pneumonia in hospitalized children based on big data analysis (9). Predictors of refractory *Mycoplasma pneumoniae* pneumonia have been studied. Studies have shown that clinically relevant risk factors for refractory *Mycoplasma pneumoniae* pneumonia are extrapulmonary complications, large area lung morphogenesis, and elevated C-reactive protein (CRP) and lactate dehydrogenase (LDH) levels (10, 11).

However, there are currently no predictive methods for severe adenovirus pneumonia. In some studies, the results were complications from severe adenovirus pneumonia rather than predictors (3, 5, 6). Early and accurate identification, treatment, and prognosis of children with adenovirus pneumonia have positive significance.

To develop a scale for the early prediction of severe adenovirus pneumonia in hospitalized children, we analyzed the clinical data of 132 children with adenoviral pneumonia admitted to the Children's Hospital Affiliated with Nanjing Medical University in the past three years and provided evidence-based medical opinions.

## 2. Materials and methods

### 2.1. Ethics

The study was approved by the Institutional Ethics Committee of the Children's Hospital Affiliated with Nanjing Medical University (approval number: 202205067-1). All procedures were performed in accordance with the principles of the Declaration of Helsinki.

### 2.2. Patients and grouping

**Figure 1:**
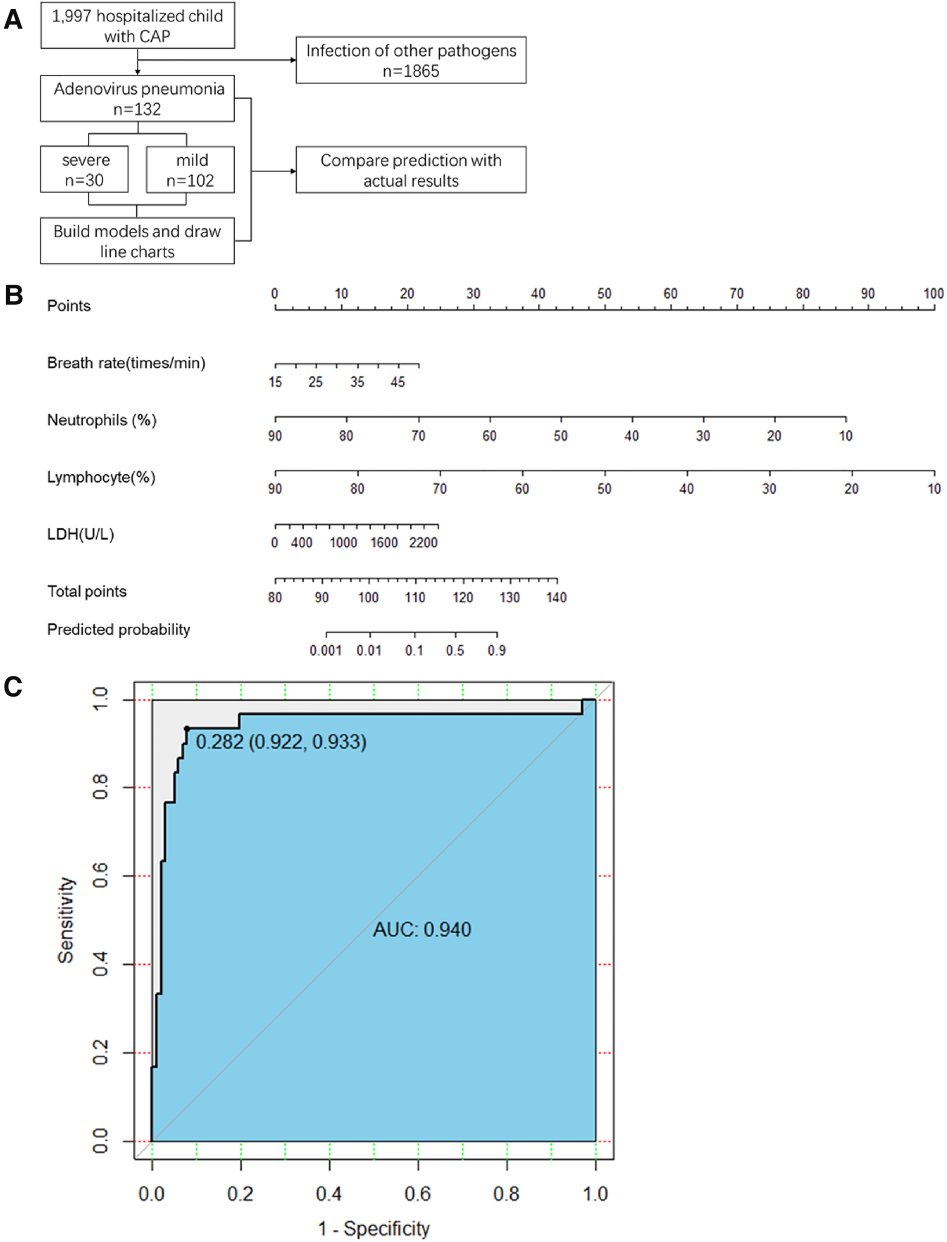
(**A**) Study flow. CAP, community-acquired pneumonia (**B**) The first line is the score corresponding to each indicator value. The following lines correspond to the indexes included in the scale, calculated total score, and predicted probability. When used, the table should be scaled up and printed on paper, and the score should be calculated using a tool such as a ruler. LDH, lactate dehydrogenase (**C**) Scale for predicting severe adenovirus pneumonia by receiver operator characteristic curves in the retrospective cohort. AUC, area under the curve.

A flowchart of the study is shown in Figure 1A. This retrospective observational study was conducted at the Children's Hospital of Nanjing Medical University from January 2017 to December 2019. This was followed by a prospective cohort study conducted at the Children's Hospital of Nanjing Medical University. Adenovirus infection was confirmed by polymerase chain reaction testing of nasopharyngeal swab specimens. The inclusion criterion was no severe pneumonia on admission.

Children with a clinical diagnosis and radiographic findings of pneumonia were included. All patients received homogenized treatment. Children with a history of pneumonia within 28 days before admission and those in the convalescent stage of pneumonia were excluded. According to the nasopharyngeal suction adenovirus nucleic acid detection results, patients were divided into two groups: adenovirus-positive and adenovirus-negative groups.

The severe group standard included patients with clinical manifestations of adenovirus pneumonia who met at least one of the following signs: disturbance of consciousness, oxygen saturation < 92%, marked shortness of breath (respiratory rate > 70 breaths per minute in infants and 50 breaths per minute in older children), dyspnea cyanosis, extrapulmonary complications, refusal to eat, dehydration, and severe chest imaging findings (pneumothorax, pleural effusion, atelectasis, or lobular infiltration).

### 2.3. Data collection and study variables

We collected data on demographic and clinical characteristics, including sex, age,, fever duration before admission, chest imaging findings, presence of underlying diseases, the respiratory rate on admission, laboratory test results, including routine blood tests (white blood cells, % neutrophils, the absolute count of neutrophils, % lymphocytes, CRP and biochemical blood indicators [aspartate aminotransferase, alanine transaminase (ALT), and LDH], and the etiology of available test results. After the preliminary screening of all indicators, statistically significant indicators were selected for regression analysis.

### 2.4. Respiratory pathogens

An adenovirus nucleic acid detection kit was used to detect nasopharyngeal aspiration in the children within 24 h of admission. Nucleic acids were extracted according to the instructions for adenovirus nucleic acid detection, and the conserved region was amplified by Q-PCR. The conserved region was compared to the standard curve and identified using agarose gel electrophoresis.

### 2.5. Statistical analysis

Epidemiological data were analyzed using SPSS Version 25.0 (IBM Corp, Armonk, NY, USA) and R Version 4.2.1 (R Foundation for Statistical Computing, Vienna, Austria). *P* < 0.05 was considered statistically significant. Normally distributed continuous data were analyzed using t-tests, and non-normally distributed measurement data were analyzed using Mann–Whitney U tests.Different populations were compared using the chi-squared test. A multivariate analysis was performed using a stepwise logistic regression model. The R software was used to transform the final regression model into a nomogram. Receiver operating characteristic (ROC) curves were used to analyze the regression model for predicting severe adenovirus pneumonia. The sensitivity and specificity of the predictive scale were calculated.

## 3. Results

### 3.1. Patient characteristics and laboratory findings

We retrospectively enrolled 132 patients with non-severe adenovirus pneumonia. Of these, 30 patients developed severe adenovirus pneumonia, and 102 patients had mild adenovirus pneumonia. The characteristics of patients in the retrospective cohort on admission are summarized in Table 1. There was no significant difference in sex distribution or radiographic chest pneumonia between the two groups. The average age, breathing rate, and fever duration before admission were significantly greater in the critical adenovirus pneumonia group than in the mild adenovirus pneumonia group. Compared to mild adenovirus pneumonia, significantly more patients in the severe adenovirus pneumonia group experienced complications. Compared to the mild group, the severe group showed significantly higher levels of neutrophils, lymphocytes, ALT, and LDH. Other laboratory findings did not differ significantly between the two groups.

**Table 1:** Admission characteristics of the children in the retrospective cohorts. Values are presented as mean ± SD.

Characteristic	Mild (*n* = 102)	Severe (*n* = 30)	t/*X*^2^	*P*-value
Sex (male/female)	55/47	18/12	0.32	0.55
Age (years)	3.52 ± 0.35	2.19 ± 0.35	1.99	0.047
Breathing rate (times/min)	31.66 ± 0.46	37.27 ± 1.29	4.23	<0.0001
Fever days	7.559 ± 0.41	10.60 ± 0.82	3.46	0.0007
Chest radiograph pneumonia (yes/no)	100/2	28/2	0.25	0.8
Basis of complications (yes/no)	25/77	12/18	13.29	0.000
WBC (×10^9^/l)	9.59 ± 0.45	10.16 ± 1.12	0.5	0.57
Neutrophil (%)	50.86 ± 1.63	61.70 ± 2.70	3.17	0.001
Lymphocyte (%)	48.19 ± 1.67	30.50 ± 2.44	5.07	<0.0001
Neutrophils (×10^9^/l)	5.07 ± 0.33	6.26 ± 0.91	1.50	0.13
CRP (mg/l)	18.51 ± 2.15	27.33 ± 5.11	1.82	0.07
ALT (U/l)	42.20 ± 3.09	69.32 ± 11.66	3.21	0.002
AST (U/l)	90.96 ± 18.54	106.90 ± 18.19	0.42	0.67
LDH (U/l)	458.5 ± 31.21	908.0 ± 106.4	5.54	<0.0001

WBC, white blood cells; CRP, C-reactive protein; ALT, alanine aminotransferase; AST, aspartate aminotransferase; LDH, lactate dehydrogenase.

### 3.2. Logistic regression and nomogram

All statistically significant variables in the intergroup comparison were considered for logistic regression analysis. The maximum likelihood ratio forward stepwise regression method was used to screen variables. Finally, breathing rate, % neutrophil, % lymphocyte, and LDH levels were included in the predictive model (Table 2). The final prediction model is shown in Figure 1B.

**Table 2:** Logistic regression analysis predictors of severe adenovirus pneumonia.

Variable	*β*	SE	Wald	*P*-value	OR	95% CI For OR	
						Lower	Upper
RR	0.175	0.044	15.524	<0.0001	1.191	1.092	1.299
N%	−0.415	0.051	67.137	<0.0001	0.660	0.598	0.729
L%	−0.449	0.052	74.174	<0.0001	0.638	0.576	0.707
LDH	0.003	0.001	34.130	<0.0001	1.003	1.002	1.004

RR, respiratory rate; LDH, lactate dehydrogenase.

### 3.3. ROC curve analysis

In the retrospective cohort, the area under the curve for the predictive scale was 0.94 [95% confidence interval (CI) 0.922–0.933], as determined by ROC curve analysis (Figure 1C). The optimal cut-off of the scale for predicting severe pneumonia was 0.282, with a sensitivity of 93.3%, specificity of 92.1%, and consistency rate of 92.42% in the retrospective cohort (Table 3).

**Table 3:** Predictive results of the early recognition scale for critically ill children with adenovirus pneumonia in a historical cohort. Sensitivity and specificity were 93.3% and 92.1%, and the diagnostic consistency was 92.42%.

		Actual	
		Severe	Mild
Predicted	Severe	28	8
	Mild	2	94
Total		30	102

## 4. Discussion

In this study, based on a clinical cohort of children with CAP, 30 patients developed severe adenovirus pneumonia, and 102 patients had mild adenovirus pneumonia. There were statistically significant differences in age, respiratory rate, fever duration before admission, percentage of neutrophils and lymphocytes, ALT, and LDH between the two groups. Logistic regression analysis was conducted using the R language, and respiratory rate, percentage of neutrophils, percentage of lymphocytes, and LDH were used as scale indicators. The preliminary establishment of a critical early recognition scale for adenovirus pneumonia in children has been completed. Using the ROC curve, the sensitivity and specificity of the scale were 93.3% and 92.1%, respectively, and the critical early recognition scale was presented in the form of a rosette diagram. The scale for preliminary early recognition of severe cases has good specificity and sensitivity, which proves that it is feasible to establish a scale for early recognition of critical cases of adenovirus pneumonia in children based on big data from a clinical cohort.

Early and accurate identification of severe cases is vital for the treatment and prognosis of adenovirus pneumonia in children. Studies have shown that early administration of cidofovir is important in improving respiratory failure caused by adenovirus pneumonia (12, 13). Methods such as PSI and CURB-65 scores have been established to predict the severity and prognosis of adult CAP and guide treatment at an early stage. The PSI score scale is a common clinical CAP assessment tool that can guide the initial treatment mode and help inform patients of expected clinical outcomes (14). The CURB-65 scoring system, developed in 2003 to assess the severity of CAP and predict short-term mortality, is highly regarded by the American Society for Infectious Diseases and the American Thoracic Society. An analysis of the diagnostic efficacy of CURB-65 found that when CURB-65 was 3, the sensitivity, specificity, and area under the curve of CAP were 56%, 74%, and 0.69, respectively, indicating good diagnostic efficacy (8). However, CAP caused by different pathogens in children of different ages has distinct clinical characteristics. Establishing a predictive tool for all etiology-induced CAP in children, such as the adult PSI or CURB-65 scores, is impossible. Therefore, it is necessary to establish a simple, accurate, and quantitative early recognition scale for severe disease in children with adenovirus pneumonia to predict early and guide treatment.

Some clinical indicators of adenovirus pneumonia are correlated with prognosis. Adenovirus pneumonia in adults is usually accompanied by a decrease in peripheral lymphoid count (15), and there is a correlation between the number of monocytes in the peripheral blood of adults and the occurrence of respiratory failure in adenovirus pneumonia (16). Adenovirus viral load and type are associated with severe adenovirus pneumonia (17). However, there is a lack of a scale for the early recognition of acute adenovirus pneumonia in children, and the early recognition of acute pneumonia depends on the experience of clinicians. Therefore, it is necessary to establish a simple, accurate, and quantifiable early identification scale for severe cases of adenovirus pneumonia in children.

Various infectious diseases are most susceptible in childhood, and children soon enter a susceptible state after the temporary immunity endowed by maternal antibodies. Childhood infectious diseases have been widely prevalent throughout human history. Morbidity and mortality rates were extremely high in the early 20th century and were an important social and medical problem. With national public health initiatives, remarkable achievements have been made in preventing and controlling infectious diseases. The prevention and treatment of infectious diseases in children is still the focus of global health work in the 21st century. There are even clusters of critically ill cases in individual regions, including multiple outbreaks of adenovirus pneumonia in China, Singapore, and South Korea (18–21). Children are particularly affected by adenoviruses owing to their unique physiological characteristics. The epidemic is endemic in some parts of our country, greatly impacts children's health, and puts great pressure on the medical system (5, 22). Prevention and control measures for nosocomial adenovirus infection and treatment plans for severe adenovirus infection and adenovirus pneumonia in children are major emerging.

A clinical cohort with a longer period, scale generation based on big data, and parallel prospective validation is needed to increase the accuracy and scientificity of the scale.

In summary, we included four off-the-shelf clinical indicators for predicting severe adenovirus pneumonia. This predictive scale helps determine whether a child will develop severe adenovirus pneumonia early in the disease course. In the retrospective cohort, the scale had better discriminative power and higher sensitivity and specificity.

## Data Availability

The original contributions presented in the study are included in the article/Supplementary Material, further inquiries can be directed to the corresponding author/s.
